# Universal Principles in the Repair of Communication Problems

**DOI:** 10.1371/journal.pone.0136100

**Published:** 2015-09-16

**Authors:** Mark Dingemanse, Seán G. Roberts, Julija Baranova, Joe Blythe, Paul Drew, Simeon Floyd, Rosa S. Gisladottir, Kobin H. Kendrick, Stephen C. Levinson, Elizabeth Manrique, Giovanni Rossi, N. J. Enfield

**Affiliations:** 1 Language & Cognition Department, Max Planck Institute for Psycholinguistics, Nijmegen, Netherlands; 2 School of Language and Linguistics, University of Melbourne, Melbourne, Australia; 3 Department of Social Sciences, Loughborough University, Loughborough, United Kingdom; 4 Radboud University, Nijmegen, Netherlands; 5 Donders Institute, PB 9104, Nijmegen, Netherlands; 6 Department of Linguistics, University of Sydney, Sydney, Australia; Max Planck Institute for Human Cognitive and Brain Sciences, GERMANY

## Abstract

There would be little adaptive value in a complex communication system like human language if there were no ways to detect and correct problems. A systematic comparison of conversation in a broad sample of the world’s languages reveals a universal system for the real-time resolution of frequent breakdowns in communication. In a sample of 12 languages of 8 language families of varied typological profiles we find a system of ‘other-initiated repair’, where the recipient of an unclear message can signal trouble and the sender can repair the original message. We find that this system is frequently used (on average about once per 1.4 minutes in any language), and that it has detailed common properties, contrary to assumptions of radical cultural variation. Unrelated languages share the same three functionally distinct types of repair initiator for signalling problems and use them in the same kinds of contexts. People prefer to choose the type that is the most specific possible, a principle that minimizes cost both for the sender being asked to fix the problem and for the dyad as a social unit. Disruption to the conversation is kept to a minimum, with the two-utterance repair sequence being on average no longer that the single utterance which is being fixed. The findings, controlled for historical relationships, situation types and other dependencies, reveal the fundamentally cooperative nature of human communication and offer support for the pragmatic universals hypothesis: while languages may vary in the organization of grammar and meaning, key systems of language use may be largely similar across cultural groups. They also provide a fresh perspective on controversies about the core properties of language, by revealing a common infrastructure for social interaction which may be the universal bedrock upon which linguistic diversity rests.

## Introduction

A design requirement for a communication system with complex, varying content, is that when communication fails there should be some mechanism to ‘repair’ it. This paper investigates a key system of communication repair found in the core ecological niche for language, conversation [1,2]. We compare conversation in 12 languages from 5 continents and find a robust system for the real-time resolution of breakdowns in communication. We find that this system of *other-initiated repair* is frequently used, that its basic structure is the same across languages, and that its principles of usage reveal the fundamentally cooperative nature of human communication.

In other-initiated repair, a recipient of a linguistic message signals that there is a problem understanding or hearing what was said, and the sender then ‘fixes’ it. Aspects of this system have been described for English [3–10], but no broad-ranging, systematic cross-cultural comparison has been made.

Comparative work is important for two reasons. First, methods for recovery from communication problems vary radically across species. Non-human animal communication systems feature re-doings, detection of unreliable signals, and failures of communication being allowed to stand or inferred later [11–14], but there appear to be few if any mechanisms for the interactive recognition and repair of breakdowns. If cross-cultural investigation revealed a basic set of mechanisms for interactive repair in human language, this would shed new light on human capacities for language, and provide a key point of comparison for the cross-species ethology of communication.

A second reason for systematic comparison is the common assumption of cross-cultural variation within our species: “While clarification is a universal activity, the manner in which clarification is accomplished varies crossculturally” [15,16]. Different languages may offer different ways to solve communication problems; or there may be a common toolbox of techniques, with not all languages using all of the tools. Work in interactional linguistics has suggested that in the domain of self-initiated repair, interactional practices are constrained by the syntactic organisation of a language [17]; this raises the question to what extent strategies for other-initiated repair may be language-specific. Yet there are also arguments in favour of a universal system. While languages may vary in fundamental ways, from sound systems to syntax to semantics [18,19], recent work has shown robust universal features in the basic infrastructure for social interaction, for instance the turn-taking system [20,21]. Likewise, practices of other-initiated repair may be so crucial to the organisation of social interaction and the achievement of joint goals that there remains little room for radical cross-cultural variation [1,2,22,23]. As one account proposes, “It is hard to imagine a society or culture whose organization of repair does not include a repair component, and one that works more or less like the one I have sketched” [1].

This generates two opposing hypotheses: a *pragmatic diversity hypothesis*, by which systems of language use reflect cultural differences and therefore may vary across cultural groups (implying or at least allowing universality in other areas of language such as grammar); and a *pragmatic universals hypothesis*, by which systems of language use are largely similar across cultural groups (allowing diversity in other areas of language) [23]. Here we test these opposing hypotheses in the domain of other-initiated repair. We also test the cross-linguistic generality of two existing proposals about repair. The first is an ordering of repair initiation techniques from ‘weak’ to ‘strong’ [3], according to which participants prefer more specific repair initiation techniques like ‘Who?’ over less specific ones like ‘Huh?’ when they can: the ‘strongest initiator rule’ [24]. The second is a principle of least collaborative effort, according to which the selection of repair initiation techniques would be done in such a way that it minimizes joint work [24,25]. Both proposals have been put forward on the basis of English data; our cross-cultural study allows us to test whether they apply in conversation across languages.

## Materials and Methods

We built video corpora of maximally informal social interaction from 12 languages of 8 language families spoken on 5 continents (Table 1). The languages vary fundamentally in typological profile (e.g., sound structure, word order, and grammatical systems), semiotic modality (spoken as well as signed), and societal setting (from small-scale peasant societies to large-scale post-industrial nations). Data were collected from consenting participants in accordance with protocols approved by the ethical review board of the Seventh EU Framework (240853 HSSLU). Consent procedures were adapted to local requirements following recommended practices in anthropology and linguistics [26,27] in a procedure approved by the ethical review board: written consent for literate participants, and audio-recorded verbal consent for non-literate participants, all archived with the conversational data. Data collection was limited to spontaneous, naturally occurring conversations between families and friends, following established methods for the collection and sampling of conversational data [21,28]. Participants often engaged in additional activities during these conversations (e.g., eating, playing games, preparing food), introducing variation which we use as a lever to gauge how factors like attention influence the signalling and resolution of communicative trouble.

**Table 1:** Languages and researchers involved in this study.

Language	Language family	Location	Researcher
Cha'palaa	Barbacoan	Ecuador	Simeon Floyd
Dutch	IE (Germanic)	The Netherlands	Mark Dingemanse
English	IE (Germanic)	United Kingdom	Kobin H. Kendrick
Icelandic	IE (Germanic)	Iceland	Rósa S. Gísladóttir
Italian	IE (Romance)	Italy	Giovanni Rossi
Lao	Tai	Laos	N. J. Enfield
Argentine Sign Language (LSA)	Italian Sign Language	Argentina	Elizabeth Manrique
Mandarin	Sinitic	Taiwan	Kobin H. Kendrick
Murrinh-Patha	Southern Daly	Northern Australia	Joe Blythe
Russian	IE (Slavic)	Rusland	Julija Baranova
Siwu	Kwa	Ghana	Mark Dingemanse
Yélî Dnye	Isolate	Island Melanesia	Stephen C. Levinson

For each language, researchers collected, transcribed, and coded around 4 hours of spontaneous conversation, resulting in 50 hours of directly comparable corpus material.

**Fig 1:**
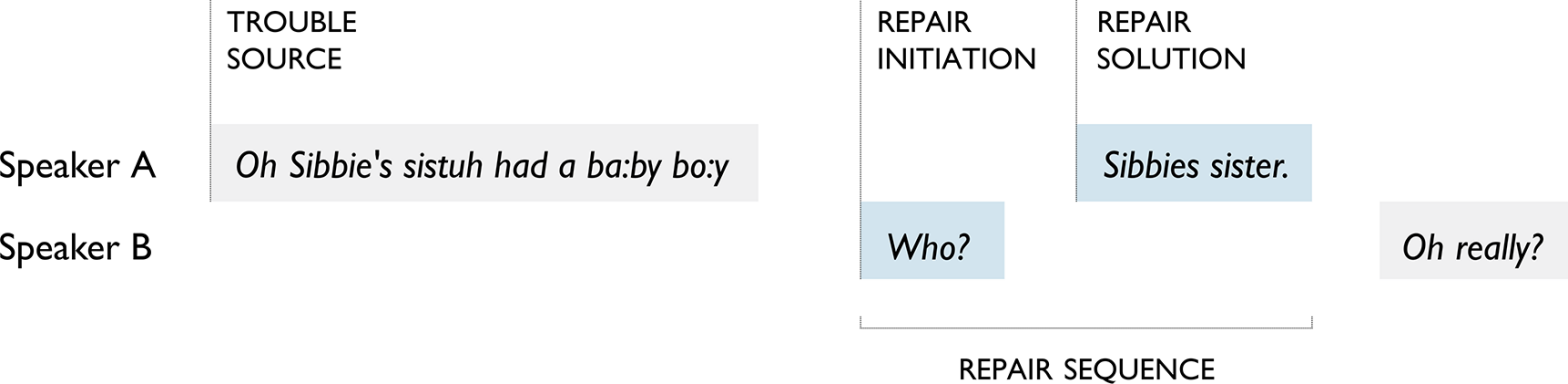
Elements of other-initiated repair. Repair sequences consist of a repair initiator that points back to a prior turn (trouble source) and points forward to a next turn (repair solution) [3].

Other-initiated repair is done in a question-and-answer type exchange that briefly disrupts the progress of an interaction. We focus on the following elements of this system and their relations to each other (Fig 1): repair initiator, a signal from speaker B of a problem with what speaker A just said, which A should fix; repair solution, how the problem is fixed, e.g., by A repeating the trouble source turn or part of it, by specifying something that was vague or missing, or by confirming that a solution proposed by B was the right one; and repair sequence, a side sequence [4] consisting of repair initiator and solution taken together. Throughout, B refers to the person initiating repair and A to the original speaker and provider of the repair solution.

We systematically sampled the conversations for occurrences of other-initiated repair, taking 10-minute segments from as many different interactions as possible to ensure against any bias from over-representation of particular interactions or speakers. Based on close analysis of other-initiated repair sequences, a cross-linguistic coding scheme was developed and applied [29,30]. The coding scheme captured facts about linguistic resources (e.g., interjections, question markers, repetition, confirmation), conversational sequence (e.g., whether the repair initiation was the first or a subsequent attempt at resolving the trouble, whether the turn preceding the repair initiation was a question or an answer), and environmental and attentional factors (e.g., whether there was auditory or visual interference, whether B was involved in a parallel activity). To maximise coding consistency across the individual languages, all researchers participated in the development of the coding scheme and in the calibration of joint understanding of the coding categories. We checked coding reliability for all coders based on a common English dataset. For the quantitative analyses, we consider only variables that achieved a Krippendorff’s α [31] of ≥ 0.66 or ≥ 75% agreement (we use % agreement for variables that achieved low α values due to skewed distributions, the well-known ‘high agreement, low consistency’ paradox [32,33]). Two variables were recoded using a narrower coding instruction. In addition to the coded variables, our quantitative analyses use 13 automatically calculated measures like absolute and relative length of elements of the repair sequence, Levenshtein distances, source recording, language, language family, etc. (as detailed in S2 Text). Data were analysed using linear mixed effects models [34] for maximal statistical power, while controlling for historical relations between languages (Galton’s problem [35]) and other dependencies and imbalances in the data. Examples of basic repair initiator types in all the languages are given in S1 Text. Details about data structure and models are provided in S2–S6 Texts. Reported statistics come from mixed effects model estimates unless otherwise noted.

## Results

Our findings fall into two rubrics: the basic properties of the system and the principles of its use. Both are loci for potential cultural and linguistic variation.

### Other-initiated repair is frequent

**Fig 2:**
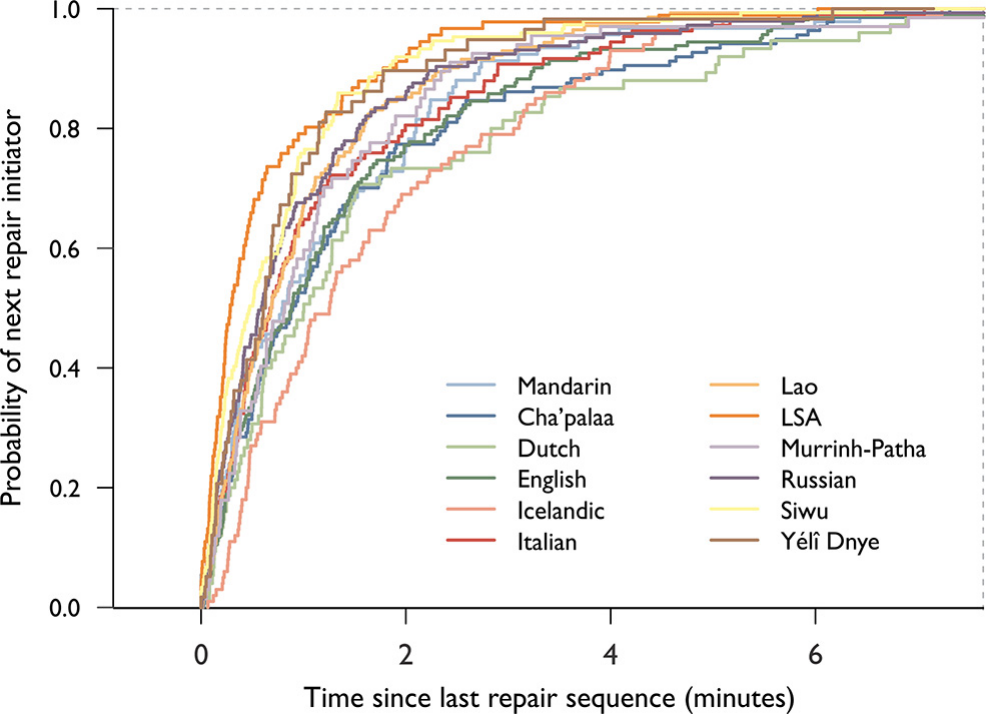
The frequency of repair. Empirical density curve showing the proportion of independent repair initiations encountered after a given amount of time has elapsed since the last one. The vast majority of repair initiations happen within 5 minutes of each other.

Other-initiated repair occurs in all of the languages in our sample. In the 48.5 exhaustively sampled hours of conversation we find 2053 cases, meaning that there is a repair initiation about once every 1.4 minutes across all languages. Counting only independent sequences, 95% of repair initiations happen within 4.13 minutes of the last one (Fig 2)—in other words, on average, no five minutes go by in any of the languages without there being a sequence of other-initiated repair. This shows that repair is a fundamental and frequent feature of conversation everywhere.

### All languages share three basic types of repair initiator

Three main practices for repair initiation recur across all of the languages in our sample: (I) open request signals a problem with the trouble source while leaving open where or what it is, and requests clarification (example: ‘Huh?’); (II) restricted request requests specification or clarification of a specific component of the trouble source (example: ‘Who?’); (III) restricted offer offers a candidate for what was just said and asks for confirmation (example: ‘she had a boy?’) [29]. These repair initiator types go from least specific (open request) to most specific (restricted offer) in terms of the amount of information they contain about the communicative trouble and the possible solution. They differ in terms of how the repair initiator targets trouble in the prior turn (open vs. restricted [6]) and what kind of response is relevant in the next turn (request vs. offer [36]).

In each of the languages, the three basic types make up the vast majority of cases of other-initiated repair (mean 92% σ 4.5%), and they are implemented using similar linguistic resources: interjections, question markers, prosody, and repetition [37,38] (see S1 Text for examples). Repetition in particular is common in repair initiators: across all languages, 48.3% of repair initiator tokens feature partial (42.6%) or full (5.7%) repetition of the trouble source turn, pointing to the importance of other-initiated repair as a mechanism for achieving interactive alignment [39–42]. We measured the length of repair initiators in orthographic characters, a commonly used measure [43] which is highly correlated with length in phonemes and with turn duration (S6 Text). The three types of repair initiator differ in their mean lengths: open = 3.7, restricted request = 10.4, restricted offer = 13.0. The differences between these lengths correlate with other elements of the repair sequence in ways that are not statistically different across the languages (S3 Text); for instance, the length of the repair solution relative to the trouble source differs significantly by repair initiator type, as shown below. Basic types may be combined into more complex formats (8% of cases across all languages), and language-specific details may offer special affordances for initiating repair [29,44,45], yet the cross-linguistic diversity appears to be constrained by universal dimensions of the system uncovered here.

In sum, all of the languages in our sample share a basic inventory of techniques to initiate repair, and the three basic types differ from each other formally in similar ways across the languages in our sample.

### Basic types behave the same

Each type of repair initiator can be thought of as inviting a repair solution that carries out a distinct kind of *operation* by the original speaker on the trouble source turn. According to qualitative studies of other-initiated repair, for open request type the canonical operation involves repetition, for restricted offer type it is a confirmation—e.g., ‘Yeah’—and for restricted request type it may be repetition or clarification [3,6,46,47]. This predicts measurable differences in the relative length of repair solutions: longest for open request, shortest for restricted offer, and in between for restricted request type. We found that the average length of the repair solution relative to the trouble source decreases as the repair initiator goes from open type to restricted request type to restricted offer type (χ^2^ = 50.7, p < 0.00001). This pattern does not differ significantly across languages (S2 Text).

In sum, repair initiator types acts as an ‘instruction’ for the repair solution in ways that are similar across languages, and they can be ordered open request < restricted request < restricted offer in terms of how the repair solution differs from the trouble source.

The above findings show that the basic properties of the system for other-initiated of repair are similar across the languages in our sample. We now consider whether there might be cross-cultural variation in when and how people use these practices.

### Specificity principle

When people initiate repair, they must choose from among less or more specific types of repair initiator. This choice could be culturally variable or more universally systematic. We first try to determine the conditions of usage of the open repair initiator. We hypothesized that open type repair initiators (such as ‘Huh?’ and ‘Sorry?’) are most likely in contexts that are particularly prone to trouble in hearing or processing what someone just said or signed [6,24,48]. These *trouble-prone contexts* were coded for in each repair sequence in terms of interference to the signal (noise or overlap), distractions to B’s attention (parallel activity), and status of the trouble source turn as an answer versus a question (answers being easier to anticipate and thus easier to process).

**Fig 3:**
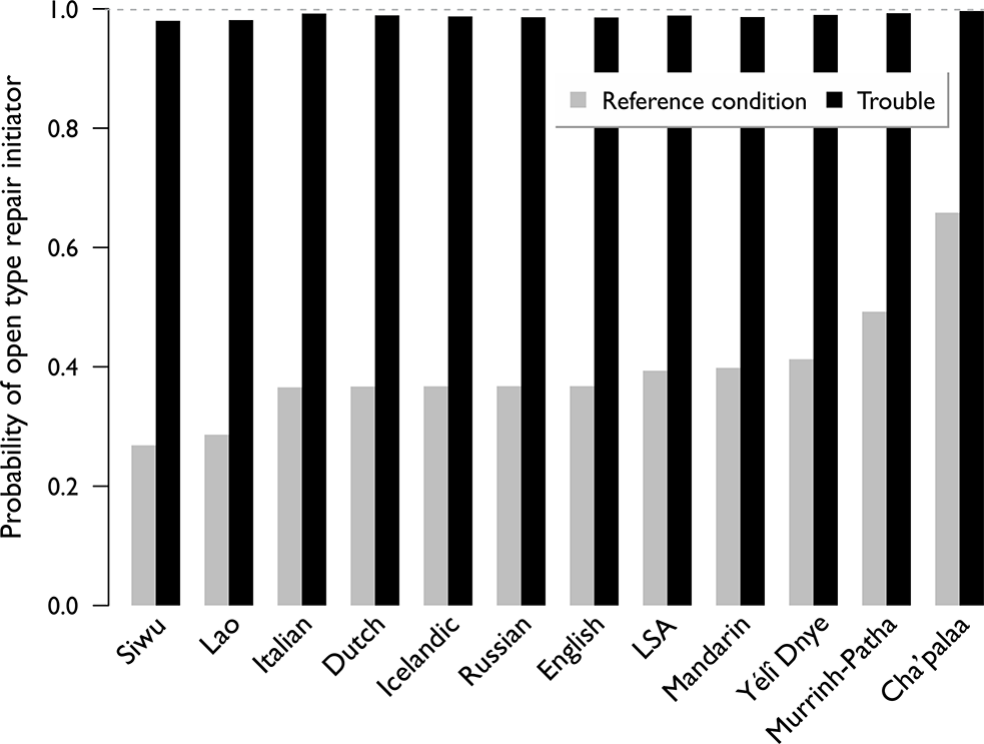
The probability of Open repair initiators in different conditions. Model estimates of the probability of Open repair initiator in reference condition (grey) versus when all three measures of trouble-prone contexts are true (black). In the latter case, probability of an Open repair initiator approaches 1 in all of the languages.

We find that in all the languages, trouble prone contexts make open type initiators more likely (χ2 = 1266.4, df = 101 p < 0.000001; S2 Text). When all three measures of trouble-prone contexts obtain, the relative probability of an open repair initiator approaches 1 in all of the languages (Fig 3). The probability that an open type is used rather than a restricted type is not affected by other variables such as speaker or recipient gaze, modality of the language being spoken, or controlled nature of the conversational setting. While languages may vary in the relative frequency with which their speakers use open type versus restricted type repair initiators (S2 Text), we find that the same contexts affect the relative probability in the same way across all languages, painting a picture of potential diversity constrained by universal principles.

The estimated probability of an open type repair initiator (P_open_) in non-trouble-prone contexts is 39% (S2 Text). Open type repair initiators are significantly more likely to be used if (a) there is *noise interference* during the trouble source turn (P_open_ = 78%, p < 0.0001); if (b) there is *overlap interference* during the trouble source turn (P_open_ = 76%, p < 0.0001); or if (c) B is in a *parallel activity* during the trouble source turn (P_open_ = 76%, p < 0.0001). Open type repair initiators are *less* likely to be used if (d) trouble source is an *answer as opposed to a question* (P_open_ = 9%, p < 0.0001); or if (e) there is *intervening material* between the trouble source and the repair initiation (P_open_ = 16%, p < 0.0001); or if (f) the *trouble source is relatively long* (P_open_ = 31%, p < 0.0001). The lower likelihood in (e,f) may be due to the fact that open repair initiators normally rely on contiguity to the prior turn to signal trouble [4,49] (and see S2 Text), and to the recipient having potentially had more time to process prior talk.

Earlier observations based on English have shown that when other-initiated repair does not immediately lead to a satisfactory solution but instead goes another round, people tend to become more specific in their subsequent choice of repair initiator [3,24]. We looked at complex sequences that feature multiple subsequent attempts at repair initiation and confirm that this tendency holds across all the languages in our sample: there was a significant interaction between the status of a repair initiator as first versus subsequent in complex sequences and the previous repair initiator type (S2 Text). Within these complex sequences, the probability of using an open type repair initiator decreases if the previous initiator was open type (P_open_ = 17.4%, z = 2.78, p = 0.0054) or restricted (P_open_ = 19.9%, z = 2.9, p = 0.0043). No other factors significantly affected the likelihood that one type of repair initiator would be chosen over another.

The findings so far point to a common principle of specificity: people choose the most specific repair initiator possible, and the choice is affected by the same kinds of factors in the same way. This is a non-trivial result: the locally egocentric behaviour for a person performing other-initiated repair would be to choose the simplest form possible (e.g., ‘huh?’) and let the other do most of the work. The principle of specificity is in line with the ‘strongest initiator rule’ and with the orientation to minimise collaborative effort known from work in the psychology of dialogue [50,51], and reveals an element of altruism in how people initiate repair in conversation. We now consider the consequences of initiating repair in terms of cost and division of labour for the participants.

### Conservation principle

When repair is initiated, part or whole of the trouble source turn is effectively lost due to the communicative trouble, and must be recovered in the repair sequence. What is the cost of these momentary disruptions to the progression of the conversation?

**Fig 4:**
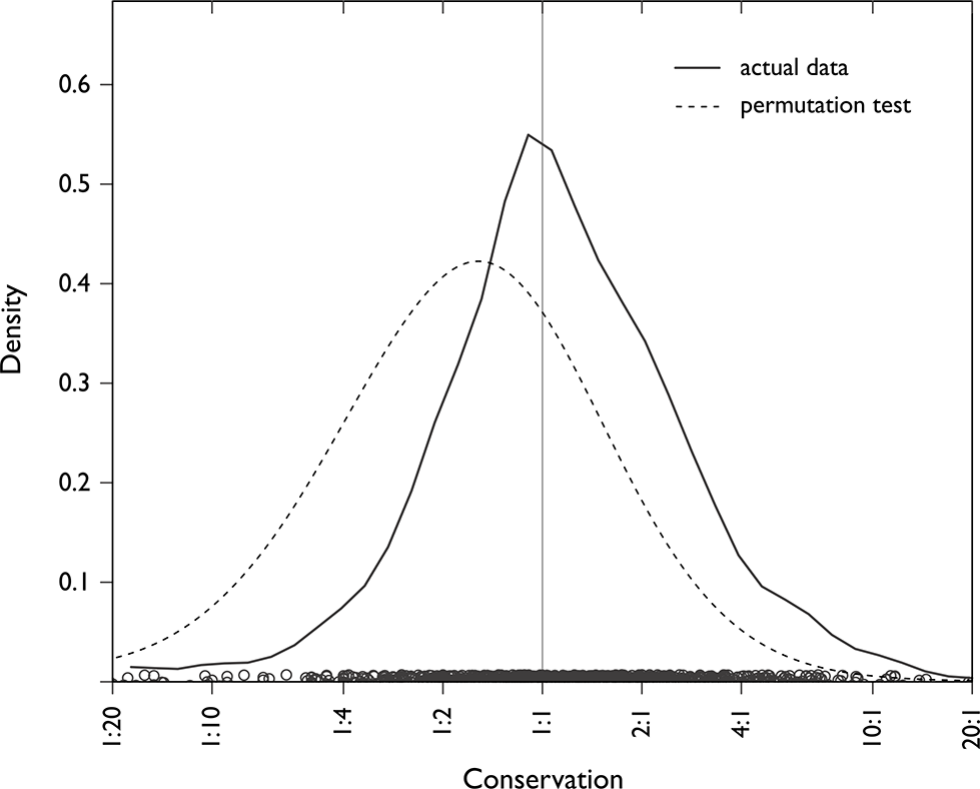
Conservation principle. Density plot of actual conservation ratios of each case in the data set (black line), with an average near 1:1; and of conservation ratios from a permutation test using randomly chosen trouble source turns (grey line), with an average of 1:1.7, closer to a null hypothesis for conservation (simulation and further explanation in S4 Text). On average, the length of the two-turn repair sequence matches the length of the trouble source turn.

We operationalized a measure of conservation as the ratio of the length of the trouble source (spoken by A alone) compared to the length of the repair sequence (repair initiator plus repair solution, spoken by B and A taken together—see Fig 1). Given that this measure compares the length of a single turn with the length of a two-turn sequence, a null hypothesis would be that the ratio should be 1:2. But in fact, it is much closer to 1:1: on average, for independent repair sequences, the length of the two-turn repair sequence is about the same as the length of the one-turn trouble source turn (model estimate 1.2:1, 95% confidence intervals = 1.02:1, 1.47:1). This is significantly closer to 1:1 than would be expected by chance as assessed by a permutation test, (p < 0.0001, Fig 4 and S4 Text), and it is invariant across languages and across repair initiator types.

This is evidence for a principle of conservation: the shared cost of repair is no more than the lone cost incurred in the trouble source turn. In effect, this is achieved by participants doing joint work to preserve content and form where possible. For instance, in a trouble-prone context, B may have little choice but to use an open repair initiator (e.g., ‘Huh?’), and the canonical response is then for A to repeat the turn in full. In contrast, a restricted repair initiator (e.g., ‘Who?’) communicates grasp of everything that was said except the problematic part; just this part is then supplied in the repair solution (e.g., ‘Sibbie’s sister’ in Fig 1). This is another example of the principle of least collaborative effort, and its effect is that repair sequences are efficient and cost-conserving.

### Division of labour principle

**Fig 5:**
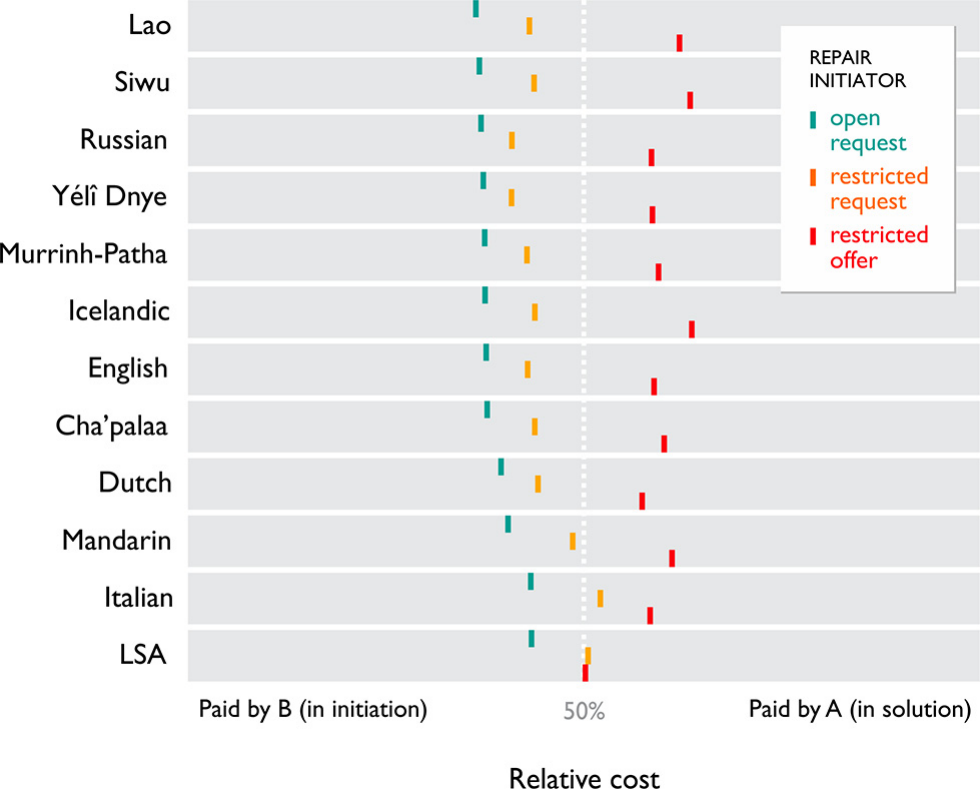
Division of labour in repair sequences. Estimated average relative costs paid by B (left of mark) and A (right of mark) for different repair initiator types are similar in each language. B pays more of the cost as repair initiators become more specific.

In other-initiated repair, a repair sequence taken as a whole constitutes the complete signalling and resolution of the communication problem at hand. How is the labour divided across participants? We measured the length of the repair sequences in our corpus, and the relative contributions by A and B to each sequence. We found that repair initiator type predicts the proportional cost that B pays (χ^2^(2) = 74.4, p < 0.0000001, model comparison: log likelihood difference = 10.4, χ2(2) = 20.9, p = 0.00003). This was invariant across languages (log likelihood difference = 5.4, χ2 = 10.85, df = 17, p = 0.86). The trend is the same for all languages: B pays more of the cost as the repair initiators become more specific, from open request to restricted request to restricted offer. This is shown by the estimated mean division of labour patterns for each language (Fig 5 and S5 Text).

The data support a principle of division of labour: across languages, the cost of doing repair is shared by B and A in a way that is predicted by the type of repair initiator. This provides additional support for the finding that the three basic repair initiator types behave the same across languages. Since the repair initiator is chosen by participant B according to the principle of specificity, the effect is that B pays as much of the cost as B can. Thus, B’s displays altruistic behaviour in selecting the most specific repair initiator possible leads to minimal joint cost.

## Discussion

Our findings offer support for the pragmatic universals hypothesis: while languages may vary in the organization of grammar and meaning, key systems of language use may be largely similar across cultural groups. The pragmatic universals we have found reveal remarkable unity where prior work proposed cultural diversity, and provide robust cross-cultural support for proposals hitherto founded only on English data [3,24]. In particular, our results provide a strong empirical verification of the cross-cultural relevance of the strongest initiator rule and the principle of least collaborative effort in conversation [24,50]. The sheer frequency of other-initiated repair (about once every 1.4 minutes across all the languages) brings home the fundamental importance of this system to human communication. The properties of the system (with three basic types accounting for the vast majority of repair initiations across languages) uncover linguistic universals of a kind not described before. The three principles of specificity, conservation, and division of labour reveal a common element of prosociality underlying the operation of the repair system in all of the languages.

Although methods to recover from breakdowns may seem essential to any communication system, things could have been otherwise. In many animal communication systems, robustness in the face of signal unreliability is provided by such properties as redundancy, multi-modality, repetition, and exaggerated or costly signals [52,53,13], all features also found in human language. Yet no other animal communication system appears to offer the kind of mechanisms for the interactive resolution of trouble we find in other-initiated repair. Our finding that it is a core feature of all 12 languages in our sample thus constitutes a substantial universal of human language, and points to the uniquely human sociality that underlies it. Another sense in which things could have been otherwise is in the distribution and use of strategies for the other-initiation of repair. It is conceivable that there are languages in which speakers use a form like ‘Huh?’ exclusively, eschewing more specific alternatives, perhaps similar to the situation in which some languages lack counting words beyond ‘one’, ‘two’, and ‘many’ [54]. Yet we haven’t found such a language; we find instead that the three basic types—open request, restricted request, and restricted offer—are used in the same situations across all the languages in our sample, showing remarkable convergence in systems of language usage and again pointing to the cooperative properties of human communication.

Our findings are based on conversation, the core ecological niche for language. As such, they provide a baseline for future work on the specifics of the repair system in different settings and societies. Corpus-based studies can build on them to investigate how different communicative settings may deploy variations of the basic system [55,56], and studies of language development can examine how and in which order children across cultures master the basic types of repair initiators [57,58]. The findings also provide an impetus for experimental work on the social and contextual factors involved in repair [59,60], and for modelling work on the theoretical aspects of achieving mutual understanding [61,62]. Finally, they provide a point of reference for cross-species ethological studies of mechanisms for repairing communicative breakdowns.

Language is a form of animal behaviour, and so, one might argue, it should be studied using the tried and tested methods of ethology [63–65]: starting with the systematic field observation of natural behaviour to establish the facts, then moving to well-designed experiments, and iterating this process to refine models and theories. Curiously, over the last 50 years, observations of natural behaviour have played little role in the discipline of linguistics, due in large part to an overly narrow conception of language as the mental competence for generating sentences [66,67]. Here we have shown that the systematic observation of language usage reveals complex phenomena such as repair that are clearly fundamental to human language, and that are tied to our uniquely human sociality.

The system for other-initiated repair described here is particularly significant for showcasing three core elements essential to human language. The first element is the property of self-referentiality (or reflexivity) in signalling: the possibility of a communication system being used not only for communicating about objects and entities in the physical world, but also for communicating about itself. Repair initiators like *Huh*?, *What did you say*?, *Who*?, *You mean John*?, as well as those repeating all or part of the prior turn, are specifically designed for drawing attention to particular elements of the communication system as it is used. Self-referentiality is a hallmark of human language [68–70], and its concrete and common use in other-initiated repair may well provide one of the main drivers behind its adaptive value in natural language.

The second element is our species’ possession of a full-blown theory of mind [71,72]: a degree of social intelligence that allows and motivates individuals to finely monitor discrepancies in states of knowledge and understanding between self and others. Conversational repair is one of the places where speakers’ theories of mind come to the surface [73], and the mechanisms of other-initiated repair offer a universally shared set of tools for the interactive achievement of mutual understanding. Third is our species’ unique capacity for cooperative and collaborative action [74,75], whereby two or more individuals can jointly commit to a shared course of behaviour, being thereby morally accountable for the success of that course of behaviour. We see these prosocial motives in action in the sequences of other-initiated repair, insofar as these sequences would not be possible without (i) fully-fledged cooperation, (ii) a willingness to delay current line of joint action and assist the other party, and (iii) a capacity to suspend and then resume the current line of action (which requires inhibiting current goals and stacking reasons for action). The cross-culturally common properties of other-initiated repair make it one of the most vivid demonstrations of the ultrasocial nature of humans [76,77].

## Conclusions

We have shown strong and systematic similarities in the properties and principles of use of a system for other-initiated repair in a diverse sample of languages, controlling for situation types, historical relationships and a range of other variables. While linguistic details of repair initiators can vary from language to language, both the general shape of the system and its principles of use in informal conversation are strongly similar across different languages, suggesting that we are tapping into the very infrastructure for social interaction [2]. These findings direct our attention to the fundamentally social nature of language. Contrary to common expectations in theoretical linguistics about the chaotic and degenerate nature of language usage [66,67], we find strong regularity and normativity in conversation, down to the level of how problems are signalled and solved.

The possibility of a universal system for other-initiated repair is important for current controversies about the essence of human language [78–80]. While those debates have focused on word- and sentence-level features like sound systems and grammatical structures, here we propose language universals in the patterns of conversation. This presents an opportunity for progress in answering the question: If language is universally and quintessentially human, what is at its core? The repair system we observe is one of the crucial safeguarding mechanisms for coherence in social interaction. It exhibits and exploits three elements that are crucial to human language and arguably unique to our species: self-referentiality, social intelligence, and collaborative action. If there is a universal core to language, these are the kinds of things it is made of.

## Supporting Information

S1 Data Dataset underlying the findings. (CSV)

S1 Text Repair sequences and initiator types. (PDF)

S2 Text Main model: Open and Restricted repair initiators. (PDF)

S3 Text Operation model. (PDF)

S4 Text Conservation model. (PDF)

S5 Text Division of labour model. (PDF)

S6 Text Estimating utterance length. (PDF)
