# Supplementary material for: Universal Principles in the Repair of Communication Problems
Source: PLoS One. 2015 Sep 16;10(9):e0136100. doi: 10.1371/journal.pone.0136100 (PMC4573759; doi:10.1371/journal.pone.0136100)
Supplement: S1 Text — (PDF) [file pone.0136100.s002.pdf]

Supplementary Information 1
Repair sequences and initiator types

Repair initiators can differ in several ways according to what linguistic resources they use and how they relate to prior and next turns [9, 10]. Across all languages investigated, we find three basic types of repair initiators: the open request type indicates some trouble in a prior turn and requests repetition or clarification while leaving open what or where the problem is; the restricted request type restricts the problem space by targeting some specific component of the trouble source turn and requesting clarification; the restricted offer type restrictes the problem space by targeting some specific component of the trouble source turn and offers a candidate solution for confirmation or correction. The three basic types of repair initiators are here exemplified for each of the 12 languages in our worldwide sample.

These simplified transcripts are for illustrative purposes only and do not represent timing, overlap, and other aspects of delivery. Results reported in the study are based on more detailed representations and additional coding of many properties and factors not represented here, including visible bodily behaviour, noise, speech overlap, parallel activities, attention, and participation framework (see S2-6). Detailed information on the linguistic formatting and interactional properties of other-initiated repair in these languages can be found in a special issue of Open Linguistics [5].

Table S1: Repair sequences and initiator types in 12 languages

Language (contributor)	OPEN REQUEST	RESTRICTED REQUEST	RESTRICTED OFFER
Cha'palaa<br>(Floyd)	*demanperee yumaa*<br>Turn {it} off now<br>***mm***<br>**mm?**<br>*cocina demanperee*<br>Turn the stove off.	*apao, aamama' junu kaa chipijcha yumaa llundetsunkai*<br>father, there by grandma the madroñas are getting ripe<br>**nukaa**<br>**where?**<br>*enu aamama' junu ((pointing))*<br>here by grandma	**weemujtusha, santsa mar- santa mariyasha**<br>in other places, in Santsa Mar- Santa Maria they say they are buying them at a dollar and a half<br>***putee***<br>**baskets?**<br>*jee*<br>yes
Dutch<br>(Dingemanse)	*ik ga alvast de macaroni in doen*<br>I'm going to prepare the macaroni<br>***hm?***<br>**hm?**<br>*ik ga nu alvast even de macaroni in doen*<br>now I'm going to prepare the macaroni	*Met Jan Boon toch ook?*<br>With Jan Boon too, right?<br>***wie?***<br>**who?**<br>*Jan.*<br>Jan.	*nee, we moesten zeventig minuten wachten*<br>no, we had to wait for seventy minutes<br>***zeventig?***<br>**seventy?**<br>*ja*<br>yes
English<br>(Kendrick)	Josh, were you drunk last night?<br>**What?**<br>Were you drunk last night?	Like do you see the wall,<br>**Which wall?**<br>That wall.	So it's going to be interesting to see what people put down for like a white canvas.<br>**Wha- oh what. so the people in the audience are gonna bid for it.**<br>Apparently.
Icelandic<br>(Gísladóttir)	*hvað var að í auganu*<br>what was wrong with your eye?<br>***ha***<br>**huh?**<br>*hvað var að í auganu*<br>what was wrong with your eye?	*hún er að halda eitthvað svona stelpupartí*<br>she's throwing like a girl's party<br>***hver***<br>**who?**<br>*Emilía*<br>Emilia	*ég væri alveg til í að eiga sumarbústað (einhvers staðar) þarna*<br>I would be completely up for having a cottage (somewhere) there<br>***í Dýrafirðinum***<br>**in Dýrafjörður Fjord?**<br>*já*<br>yes
Italian<br>(Rossi)	*la (fa-) scade domam vera*<br>it (fa-) it expires tomorrow right?<br>***ah***<br>**huh?**<br>*la scade domam (vera)*<br>it expires tomorrow (right)?	*non ho ancora iniziato ad usarla*<br>I haven't yet started using it<br>***cosa***<br>**what**<br>*la calcolatrice scientifica*<br>the scientific calculator	*con un po' di olio è più buona se vuoi*<br>with a bit of oil it's better if you like<br>***olio***<br>**oil?**<br>*mh*<br>mh
Lao<br>(Enfield)	*qaw3 vaj4 qaw3 vaj4*<br>put {them} away, put {them} away<br>***haa2***<br>**huh?**<br>*qaw3 vaj4*<br>put {them} away	*bòò1 mii2 sùak4 vaa3*<br>Don't you have any rope?<br>***sùak4 ñang3***<br>**Rope {for} what?**<br>*sùak4 mat1 ñuung2*<br>Rope for tying up mosquitoes.	*bòò1 huu4.cak2 mong4 man2*<br>{We} didn't know where it was.<br>***daan3 nguu2***<br>**Daan Ngou?**<br>*qee5*<br>Yeah.

LSA[1]<br>(Manrique)	*FINISH ((H-Q_ER))*<br>Did you finish?<br>***((Lean forward-head_up-Q_ER))***<br>**Huh?**<br>*MONEY FINISH- ((Q_ER))*<br>{Paying the} money, did you finish?	*SEE-I BABY ((Q_ET))*<br>Have you seen the baby?<br>***HN-NO ((Q_ET Q_ER))***<br>***WHO BROTHER WHO BROTHER-H***<br>**No, of whose brother {is it}?**<br>*I BROTHER GRANDDAUGHTER*<br>Your granddaughter's brother{'s}	*I POLISH GLASS POLISH*<br>I {work} polishing glass.<br>***CARS---H ((Q_ER))***<br>**{of} cars?**<br>*((head-nod)) YES YES*<br>Yes, yes.
Mandarin<br>(Kendrick)	*nǐ yǒu gōngkè ma*<br>do you have homework?<br>***hmm***<br>**hmm?**<br>*jīn tiān yǒu méi yǒu gōngkè*<br>do you have homework today?	*nǐ zhīdào lièrén ma*<br>do you know hunter?<br>***shénme lièrén***<br>**what hunter?**<br>*mànhuà a*<br>the cartoon	*jiù dā ge jìchéngchē, bā shí ma*<br>then I took a taxi, eighty dollars<br>***nǐ shì shuō cóng gōngguǎn jiéyùn zhàn guò lái***<br>**you're saying you came from Gongguan subway station?**<br>*bú shì wǒ shì cóng kējì dàlóu*<br>no, I came from the Technology Building
Murrinh-Patha<br>(Blythe)	*kaka ngay thama*<br>{he was} my uncle, you know<br>***aa?***<br>**Huh?**<br>*kaka ngay thama*<br>{he was} my uncle, you know	*ngarra weyi kardirdi mebert;*<br>Into the {snake} hole, she used to put her hand andgrab{it/them}.<br>***thangkugu.***<br>**what ku-thing?**<br>*ku tharingkin ku::*<br>king brown(s)	*Pelenannga*<br>Soft fat<br>***pelenangga kardunukun?***<br>**Human {kidney} fat?**<br>((nods))
Russian<br>(Baranova)	*Ej skol'ka let ta?*<br>How old is she?<br>***a:?***<br>**huh?**<br>*Ej skol'ka let ta etaj?=*<br>How old is she, this one?	*Vaz'mite zaftra ejo*<br>Take here tomorrow<br>***kavo***<br>**whom?**<br>*Ta:niu*<br>Tanya.	*U ix doch eh vyshla zamuzh, radila.*<br>*i vot nada, payedut tuda*<br>Their daughter um got married, had a child and now they need, they will go there.<br>***v Aziorsk?***<br>**to Oziorsk?**<br>*v Aziorsk.*<br>to Oziorsk.
Siwu<br>(Dingemanse)	*ɔ̃ sí ɔ̃ de ɔ̃ sate si ɔ kpi*<br>she should be responsible if he dies<br>***m:?***<br>**m?**<br>*sɔ ɔ̃ sí ɔ̃ de ɔ̃ sate*<br>that she should be responsible	*Mɛ́kɛ̀lì ɔ̃*<br>It's Michael's.<br>***ǹna mɛ́kɛ̀lì:***<br>**Which Michael?**<br>*oò, fɔ ɔse- fɔ ɔse ɔbi Mɛ́kɛ̀lì.*<br>oh, your father- your father's son Michael.	*ira iwɛ̃ ɔkuti iyóo?*<br>is there still some in the house?<br>***piɔ wɔta:***<br>**purified water?**<br>*ãi.*<br>yeah.
Yélî Dnye<br>(Levinson)	*daa wa ma ngmê*<br>will they eat it?<br>***aa?***<br>**huh?**<br>*daa wa ma ngmê*<br>will they eat it?	*mu tpile daa wa ma ngmê, apii*<br>they cannot eat that thing right?<br>***ló tpile?***<br>**what thing?**<br>*mu tpile taataa*<br>that red thing	*ngm:aa kn:ââ, mgêmî ndapî.*<br>the replacement big kê shell and the marriage shells<br>***mgêmî ndapî?***<br>**marriage shells?**<br>*nyââ*<br>yes

[1] The Argentine Sign Language examples use the following abbreviated notations: H hold, HN head nod, Q_ER question marker eyebrows raised, Q_ET question marker eyebrows together.
