# Supplementary material for: Universal Principles in the Repair of Communication Problems
Source: PLoS One. 2015 Sep 16;10(9):e0136100. doi: 10.1371/journal.pone.0136100 (PMC4573759; doi:10.1371/journal.pone.0136100)
Supplement: S2 Text — (PDF) [file pone.0136100.s003.pdf]

Supplementary Information 2
Main Model: Open and Restricted repair initiators

2.1 Overall approach

The aim of the quantitative part of this paper was to estimate what factors affect how repair is done, and to test whether these factors differ across languages. The data come from samples of conversations in many different languages and cultures. In laboratory conditions, one might attempt to experimentally control the data to obtain the same number of samples from each language in each possible condition (with noise and with intervening turns, with noise and without intervening turns etc.), and with each OIR being independent from the others. Here, we use the natural control method [11] to ensure that we compare like with like (by looking at repair initiation techniques only within repair sequences); and we use the variability inherent in naturally occurring conversation to assess the relative contributions of different factors.

Despite a regime to ensure broad comparability of recordings as well as diversity in situations sampled, we know that there are imbalances and dependencies in the data. Mixed effects modelling was used to address these imbalances and dependencies. The models define the structure of the data: other-initiations of repair (OIRs) are embedded in sequences, which are embedded in recordings, which come from a specific language community. Languages are also related historically. The fixed effect factors were chosen based on a-priori predictions about what would affect the likelihood of using open versus restricted repair initiators, as well as some possible confounding factors. Because of the structure, and due to the large number of variables of interest, the resulting model is very complex. This made it intractable to exhaustively search the possible space of models to find the 'best' model. Therefore, we present results from the most conservative model possible,

in line with the approach of maximal models for confirmatory hypothesis testing [8] (though see recent challenges to this [7]). In any case, we wanted estimates for all fixed effects. To be more confident of our results, we used a significance level of 0.01 for fixed effects.

This maximally conservative approach is the most suitable for the cross-cultural conversational corpora we work with; it is much better than doing a series of individual tests for different factors, without controlling for the non-independence of the data.

Mixed effects modelling also allowed us to test whether factors that affect how repair is done differ across languages. This was done by testing whether allowing particular factors to vary by language significantly improve the fit of the model. This ability to do a quantitative, statistical test of universality makes mixed effects modelling an extremely useful framework for this study.

2.2 Data

Table 2.1 shows the list of variables in the data with descriptions of the contents.

Table 2.1: List of variable names and descriptions

Variable Name	Description
CostB.rel	Relative length of RS compared to RI
first	Is the OIR sequence the first in a sequence of OIRs, or non-first?
ID.sequence	Sequence identity
language	Language of recording
language.family	Language family of language (according to Glottolog)
modality	Modality of conversation Audible, non-Audible
oirs.per.minute	Average number of OIRs per minute for the given recording
p4	Conservsation: Relative length of trouble source to insert sequence
previous_RI_target2	The previously used type of RI (r1,r2 vs. open)
recording	Identity of the recording session
RI_Clength.logcenter	Log, centered length of RI in characters
RI_identity	RI type open, r1, r2
RI_or	Transcription of Repair initiator
RI_target2.Restricted	RI is restricted? (r1,r2 vs. open)
RI_tr	Translation of repair initiator
RS_Clength.logcenter	Log, centered length of RS in characters
RS_or	Transcription of Response
RS_tr	Translation of Response
seq_intervene	Are there intervening turns between TS and RS?
soundproof	Was the recording made in experimental conditions? TRUE,FALSE
TS_aud	Is there audio interference no,noise,overlap
TS_aud.bin	Is there audio interference no,yes
TS_Clength.logcenter	Log, centered length of TS in characters
TS_or	Transcription of Trouble Source
TS_par	Is B engaged in parallel activity? yes,no
TS_tr	Translation of Trouble Source
TS_vis	Is there visual interference? yes,no
vis_gazeAB	Does A gaze at B?
vis_gazeBA	Does B gaze at A?

2.3 Methods

Mixed effect logit modelling was used to assess the data in R [12], using packages *lme4* [13] and *languageR* [14]. The model predicts the type of repair initiator that is used given factors relating to the previous turn.

Since the data comes from real conversations, the conditions are unbalanced. In order to control for this, four types of grouping are used as nested random effects: Cases of repair are embedded in sequences (1153 sequences, a sequence may contain more than one case of other initiated repair). Each sequence is grouped within a recording session (141 recordings). Sessions are grouped into languages (12 languages). Some languages are more historically (and therefore structurally) related, so languages are grouped within language families (8 language families).

LMER models without random slopes are prone to type I errors [15, 8]. To address this, several variables were entered as random slopes by recording and by language, as the structure of the data allowed.

The intercept of the model was set to reflect the 'reference condition' - the least marked situation (determined by frequency, which matches intuition well). The reference condition is an OIR from a 1PP, 'first' sequence from a dyadic conversation in an audible language with no visible nor audio trouble, no intervening material, no parallel activity, not recorded in a soundproof booth and where B gazes to A and A gazes to B.

2.3.1 Model structure

Below is the R code for the main model structure:

```
RI_target2.Restricted~  TS_Clength.logcenter
+ previous_RI_target2:first + previous_RI_target2 +
oirs.per.minute +
seq_part +
modality +
TS_vis + TS_vis:modality +
seq_intervene +
vis_gazeBA + vis_gazeAB + vis_gazeAB:vis_gazeBA + modality:vis_gazeBA +
vis_gazeBA:first +
TS_sequence +
TS_aud +
TS_par + TS_par:first +
soundproof +
(1 + seq_intervene + TS_sequence + TS_aud + TS_par + TS_vis
+ TS_Clength.logcenter| language) + # Group by language
(1 + seq_intervene  + TS_aud + TS_par
+ TS_vis + modality + soundproof | recording) + # Group by recording
(1 | language.family) + # Group by language family
(1 | ID.sequence)
```

2.3.2 Outlier removal

Outliers were identified by Cook's distance, Hat value and Studentized Residuals from a linear regression model containing all the fixed effects above. These identify cases that have a disproportional effect on the fit of the model. 3 cases were found to be outliers and were removed from the analysis. The main results are not qualitatively different with or without these cases.

2.3.3 R code

The R code used to run the analysis is available on request (contact sean.roberts@mpi.nl).

2.4 Results

The model converged with the following fit: AIC = 1831.5; BIC =-538.96; log likelihood = 1077.9. Table 2.2 shows the estimated fixed effects, with standard errors, z-values and probability estimates for model 1. Column 2 shows the estimated probability of using an open type repair initiator in a given situation, while column 3 shows the deviation from the reference condition. The model can make predictions about combinations of factors, such as the probability of using an open type repair initiator when there is both audio noise and intervening material. However, these predictions are not linearly related to the sum of the percentage differences for each factor. Table 2.3 shows the same results, but as raw logit estimates from the model.

Table 2.2: Estimated fixed effects, with standard errors, z-values and probability estimates for model 1.

Condition	Open RI prob.		Std. Error	z value	Pr(> |z|)	
Reference condition	39.5 %		0.41	1.05	0.296	
**Change in condition**		**Change in prob.**				
TS length (length doubles)	31.88	-7.59	0.1	3.46	0.00054	*
Intervening material	16.67	-22.79	0.28	4.29	1.80E-05	*
Trouble source is answer to question (compared to question)	9.40	-30.07	0.41	4.47	7.80E-06	*
Trouble source is 'other' (compared to question)	21.27	-18.19	0.22	3.98	6.90E-05	*
Audio interference: noise	77.65	38.19	0.49	-3.42	0.00062	*
Audio interference: overlap	76.56	37.1	0.3	-5.38	7.40E-08	*
Parallel activity	77.28	37.82	0.38	-4.39	1.20E-05	*
Previous RI is restricted	35.28	-4.18	0.21	0.86	0.39	
Previous RI is open + non-first sequence	18.05	-21.41	0.39	2.78	0.0054	*
Previous RI is restricted + non-first sequence	20.40	-19.06	0.33	2.85	0.0043	*
B does not gaze at A	38.59	-0.87	0.3	0.12	0.9	
A does not gaze at B	33.27	-6.19	0.29	0.91	0.36	
Neither A nor B gaze at each other	44.65	5.19	0.42	-0.51	0.61	
Experimental setting	2.69	-36.77	1.48	2.13	0.033	.
Visual interference	37.10	-2.36	0.54	0.18	0.85	
Non-Audible modality	86.32	46.86	1.08	-2.11	0.035	.

Table 2.3: Fixed effects results for the main model. Columns indicate variable, the estimated coefficient (logit scale), the standard error, the z value and the significance.

	Estimate	Std. Error	z value	Pr(>|z|)
(Intercept)	0.43	0.41	1.05	0.2956862
TS_Clength.logcenter	0.33	0.10	3.46	0.0005374
previous_RI_target2restricted	0.18	0.21	0.86	0.3886089
oirs.per.minute	1.22	0.86	1.41	0.1588155
seq_partmulti-party	0.09	0.24	0.39	0.6978377
modalityNon-Audible	-2.27	1.08	-2.11	0.0352031
TS_visyes	0.10	0.54	0.18	0.8534999
seq_interveneyes	1.18	0.28	4.29	0.0000178
vis_gazeBAno	0.04	0.30	0.12	0.9025820
vis_gazeABno	0.27	0.29	0.91	0.3610711
TS_sequence2PP	1.84	0.41	4.47	0.0000078
TS_sequenceother	0.88	0.22	3.98	0.0000686
TS_audnoise	-1.67	0.49	-3.42	0.0006217
TS_audoverlap	-1.61	0.30	-5.38	0.0000001
TS_paryes	-1.65	0.38	-4.39	0.0000115
soundproofTRUE	3.16	1.48	2.13	0.0329978
previous_RI_target2open:firstnotfirst	1.08	0.39	2.78	0.0054426
previous_RI_target2restricted:firstnotfirst	0.93	0.33	2.85	0.0043454
modalityNon-Audible:TS_visyes	-2.45	1.60	-1.53	0.1254236
vis_gazeBAno:vis_gazeABno	-0.21	0.42	-0.51	0.6074925
modalityNon-Audible:vis_gazeBAno	-13.79	6333.30	0.00	0.9982621
firstnotfirst:vis_gazeBAno	0.09	0.65	0.15	0.8845836
firstnotfirst:TS_paryes	1.23	0.91	1.34	0.1791825

2.4.1 Random effects covariance

	(Intercept)	seq_interveneyes	TS_audnoise	TS_audoverlap	TS_paryes	TS_visyes	modalityNon-Audible	soundproofTRUE
(Intercept)	0.82	0.47	-0.12	-0.95	-0.54	0.36	-0.77	-1.90
seq_interveneyes	0.47	0.28	-0.00	-0.53	-0.18	0.35	-0.51	-1.10
TS_audnoise	-0.12	-0.00	0.45	0.21	0.97	0.86	-0.37	0.55
TS_audoverlap	-0.95	-0.53	0.21	1.10	0.76	-0.27	0.80	2.20
TS_paryes	-0.54	-0.18	0.97	0.76	2.20	1.60	-0.50	1.80
TS_visyes	0.36	0.35	0.86	-0.27	1.60	2.10	-1.40	-0.27
modalityNon-Audible	-0.77	-0.51	-0.37	0.80	-0.50	-1.40	1.30	1.50
soundproofTRUE	-1.90	-1.10	0.55	2.20	1.80	-0.27	1.50	4.70

Table 2.4: Random effects covariance by recording

	(Intercept)	seq_interveneyes	TS_sequence2PP	TS_sequenceother	TS_audnoise	TS_audoverlap	TS_paryes	TS_visyes	TS_Clength.logcenter
(Intercept)	0.00	-0.00	-0.00	-0.00	0.00	-0.00	-0.00	-0.00	0.00
seq_interveneyes	-0.00	0.04	-0.14	-0.03	-0.03	0.07	-0.01	0.17	-0.04
TS_sequence2PP	-0.00	-0.14	0.64	0.17	0.04	-0.19	0.14	-0.68	0.13
TS_sequenceother	-0.00	-0.03	0.17	0.05	-0.01	-0.02	0.05	-0.16	0.03
TS_audnoise	0.00	-0.03	0.04	-0.01	0.09	-0.12	-0.05	-0.13	0.04
TS_audoverlap	-0.00	0.07	-0.19	-0.02	-0.12	0.20	0.04	0.32	-0.08
TS_paryes	-0.00	-0.01	0.14	0.05	-0.05	0.04	0.08	-0.09	0.01
TS_visyes	-0.00	0.17	-0.68	-0.16	-0.13	0.32	-0.09	0.82	-0.17
TS_Clength.logcenter	0.00	-0.04	0.13	0.03	0.04	-0.08	0.01	-0.17	0.04

Table 2.5: Random effects covariance by language

2.4.2 Random effects

The random intercepts by recording had small effects. The effect of the mean recording intercept by language on the full model was up to 1% point difference in the probability of an open class RI.

Table 2.6 shows the random effects by language.

	(Intercept)	seq_interveneyes	TS_sequence2PP	TS_sequenceother	TS_audnoise	TS_audoverlap	TS_paryes	TS_visyes	TS_Clength.logcenter
Chapalaa	0.00	-0.01	-0.00	-0.01	0.02	-0.03	-0.02	-0.03	0.01
Dutch	0.00	-0.16	0.63	0.14	0.14	-0.33	0.06	-0.78	0.17
English	-0.00	0.12	-0.36	-0.05	-0.18	0.31	0.03	0.55	-0.13
Icelandic	-0.00	-0.01	0.11	0.04	-0.03	0.02	0.05	-0.08	0.01
Italian	0.01	-0.07	-0.27	-0.18	0.42	-0.50	-0.38	-0.19	0.10
Lao	-0.00	-0.03	0.25	0.08	-0.05	0.01	0.11	-0.20	0.03
LSA	-0.00	-0.02	0.12	0.04	-0.02	-0.00	0.05	-0.10	0.02
Mandarin	-0.00	0.38	-1.49	-0.34	-0.31	0.73	-0.18	1.81	-0.39
Murrinh-Patha	0.00	0.01	-0.09	-0.03	0.01	0.01	-0.03	0.08	-0.01
Russian	-0.00	0.10	-0.28	-0.04	-0.15	0.26	0.03	0.43	-0.10
Siwu	-0.00	-0.14	0.76	0.21	-0.01	-0.16	0.21	-0.74	0.14
Yélî Dnye	-0.00	-0.05	0.26	0.07	0.01	-0.07	0.07	-0.27	0.05

Table 2.6: Random effects by language for the model predicting open vs restricted RI

	(Intercept)
Australian	-0.40
Barbacoan	-1.08
Indo-European	0.12
LSA	0.00
Niger-Congo	0.58
Sino-Tibetan	-0.01
Tai-Kadai	0.49
Yele	-0.07

Table 2.7: Random intercepts by language family for the model predicting open vs restricted RI

2.5 Estimating probabilities for languages

The probability of using an open class RI in the default condition for a given language L was calculated in the following way. First, the intercept of the whole model was taken. Added to this estimate was the intercept for a given language, and the intercept for that language's language family (the latter was always very small). Then the mean intercept for recordings from L was calculated, weighted by the number of cases appearing in each recording. The mean intercept for sequences from L was also calculated. These two means were added to the estimate. This gave an estimate of the probability of a restricted class RI being used in an average sequence from an average recording of language L, expressed in a logit scale. This was converted to log odds, then a percentage probability.

The difference between the estimates from the intercept of the language and language family alone, and the more detailed estimate described above was up to 10% (mean 2%).

We note that languages vary in the relative frequency with which their speakers use open type versus restricted type repair initiators. The languages differ considerably in the estimated proportion of open and restricted type responses in the reference condition, ranging from Lao (26% chance of open type) to Chapalaa (66% chance of open type; see table 2.8). The estimated probabilities correlate well with the raw proportions ($r = 0.76$, $t = 3.6$, $df = 10$, $p = 0.004$). However, there are some differences. For example, Yélî Dnye has the second largest raw proportion of open type repair initiators, but it is in the middle of the distribution for the model estimates. Conversely, Murrinh-patha is in the middle of the distribution for raw proportion, but has the second highest estimated probability of using an open type repair initiator.

Table 2.8: Estimated and raw probabilities of an open class repair initiator in different languages.

Language	Estimated Probability of open	Raw probability of open
Lao	0.263	0.211
Dutch	0.298	0.165
Siwu	0.301	0.219
Russian	0.356	0.198
LSA	0.359	0.339
English	0.386	0.217
Icelandic	0.388	0.395
Italian	0.414	0.305
Yélî Dnye	0.443	0.402
Mandarin	0.480	0.317
Murrinh-Patha	0.542	0.308
Chapalaa	0.666	0.473

2.6 Universality

We can use the model above to test whether repair works in different ways for different languages. If a model which allows the slope of a variable's coefficient to vary by language significantly improves the fit of the model, then this is evidence that repair systems differ between languages.

For each variable, two models were compared. Both models had the same fixed effects, but only one included the given variable as a random slope within languages. Here, we're using the model without random slopes by recording. This is a more conservative test of universality, because there is greater leeway for the model to improve than a model that has already other fitted random effects. Model comparisons used models that were fit using maximum likelihood, and used a Bonferroni-corrected significance level of 0.0025 (20 comparisons, although the results were not qualitatively different using a significance level of 0.05).

Table 2.9: Model fit improvement by allowing coefficients to vary by language. The column 'logLikDiff' indicates the difference in log likelihood between the original model and the revised one.

Variable	Df	AIC	BIC	logLik	deviance	χ^2	χ^2 Df	p	logLikDiff
TS_Clength.logcenter	29	1160.40	1308.57	-551.20	1102.40	0.26	2	0.88	0.13
previous_RI_target2:first	41	1181.73	1391.20	-549.86	1099.73	2.94	14	1.00	1.47
previous_RI_target2	29	1160.55	1308.71	-551.27	1102.55	0.12	2	0.94	0.06
oirs.per.minute	29	1159.78	1307.94	-550.89	1101.78	0.89	2	0.64	0.45
seq_part	29	1160.58	1308.75	-551.29	1102.58	0.08	2	0.96	0.04
modality	29	1159.37	1307.53	-550.68	1101.37	1.30	2	0.52	0.65
TS_vis	29	1157.74	1305.90	-549.87	1099.74	2.93	2	0.23	1.46
TS_vis:modality	41	1180.29	1389.76	-549.15	1098.29	4.38	14	0.99	2.19
seq_intervene	29	1160.11	1308.27	-551.05	1102.11	0.56	2	0.76	0.28
vis_gazeBA	29	1160.60	1308.76	-551.30	1102.60	0.07	2	0.97	0.04
vis_gazeAB	29	1157.10	1305.26	-549.55	1099.10	3.57	2	0.17	1.79
vis_gazeAB:vis_gazeBA	41	1179.73	1389.20	-548.86	1097.73	4.94	14	0.99	2.47
modality:vis_gazeBA	41	1183.35	1392.82	-550.68	1101.35	1.32	14	1.00	0.66
vis_gazeBA:first	41	1180.74	1390.21	-549.37	1098.74	3.93	14	1.00	1.96
TS_sequence	32	1166.45	1329.94	-551.22	1102.45	0.22	5	1.00	0.11
TS_aud	32	1165.52	1329.01	-550.76	1101.52	1.15	5	0.95	0.57
TS_par	29	1159.21	1307.38	-550.61	1101.21	1.45	2	0.48	0.73
TS_par:first	41	1179.46	1388.94	-548.73	1097.46	5.20	14	0.98	2.60
soundproof	29	1160.48	1308.64	-551.24	1102.48	0.19	2	0.91	0.09

2.7 Null model

The model achieves a fit that is significantly better than a null model with no fixed effects (log likelihood difference = 633.22, χ^2= 1266.4, df = 101 $p < 0.000001$). In the reference condition, open type repair initiators (as opposed to restricted type) are estimated to be used 39.5% of the time. This is slightly higher than the raw data suggest (28%).

Table 2.10: Model improvement over null model with no fixed effects.

Model	Df	AIC	BIC	logLik	deviance	Chisq Chi	Df	Pr(>Chisq)
Null model	5	2354.4	2382.5	-1172.18	2344.4			
Main model	106	1289.9	1831.5	-538.96	1077.9	1266.4	101	< 2.2e-16 ***

2.8 Model without intervening material

The same model was run, excluding cases which included intervening material between the repair invitation and response (950 cases). There were no qualitative differences from the main model (see table 2.11).

Table 2.11: Fixed effects results for a model run excluding cases with intervening material.

	Estimate	Std. Error	z value	Pr(>|z|)
(Intercept)	0.26	0.39	0.65	0.513
TS_Clength.logcenter	0.36	0.11	3.36	0.001
previous_RI_target2restricted	0.26	0.22	1.18	0.239
oirs.per.minute	0.98	0.85	1.16	0.247
seq_partmulti-party	0.13	0.24	0.53	0.594
modalityNon-Audible	-1.85	1.06	-1.75	0.080
TS_visyes	-0.01	0.48	-0.01	0.989
vis_gazeBAno	0.26	0.34	0.77	0.440
vis_gazeABno	0.43	0.32	1.36	0.174
TS_sequence2PP	1.88	0.44	4.24	0.00002
TS_sequenceother	0.85	0.23	3.71	0.0002
TS_audnoise	-1.85	0.50	-3.69	0.0002
TS_audoverlap	-1.22	0.30	-4.04	0.00005
TS_paryes	-1.45	0.44	-3.28	0.001
soundproofTRUE	13.06	229.64	0.06	0.955
previous_RI_target2open:firstnotfirst	1.10	0.43	2.54	0.011
previous_RI_target2restricted:firstnotfirst	0.86	0.34	2.50	0.012
modalityNon-Audible:TS_visyes	-2.68	1.54	-1.73	0.083
vis_gazeBAno:vis_gazeABno	-0.50	0.46	-1.11	0.269
modalityNon-Audible:vis_gazeBAno	-8.40	394.71	-0.02	0.983
firstnotfirst:vis_gazeBAno	0.45	0.76	0.60	0.548
firstnotfirst:TS_paryes	1.48	1.22	1.21	0.225
