# Supplementary material for: Universal Principles in the Repair of Communication Problems
Source: PLoS One. 2015 Sep 16;10(9):e0136100. doi: 10.1371/journal.pone.0136100 (PMC4573759; doi:10.1371/journal.pone.0136100)
Supplement: S3 Text — (PDF) [file pone.0136100.s004.pdf]

Supplementary Information 3
Operation model

3.1 Introduction

This model investigates the relation between repair solution and trouble source as a function of repair initiator type. See the main model supplementary materials for a description of the variables and data.

The relative length of the trouble source compared to the repair solution was calculated as:

$$TS\_RS\_relLength = log\left(\frac{RS\_Clength}{TS\_Clength}\right) \tag{3.1}$$

3.1.1 Model structure

Below is the R code for the model structure:

```
TS_RS_relLength~
RI_identity +
TS_vis +
seq_intervene +
TS_aud.bin +
TS_par +
soundproof +
(1 + RI_identity + TS_vis + TS_aud.bin + TS_par+ seq_intervene| language)
+ (1 + TS_vis + TS_aud.bin + TS_par+ seq_intervene | recording)
+ (1 | language.family)
```

3.2 Results

The results for the fixed effects are found in table 3.1, and the probability estimates are shown in table 3.2. Adding a random slope for RI type by language did not improve the fit of the model (table 3.3).

Table 3.1: Fixed effects results for the operation model.

	Estimate	Std. Error	t value
(Intercept)	-1.45345	0.12075	-12.037
RI_identityR1	0.55538	0.11957	4.645
RI_identityO	0.96317	0.13532	7.117
TS_visyes	-0.11216	0.17312	-0.648
seq_interveneyes	-0.03275	0.13408	-0.244
TS_aud.binTRUE	0.22501	0.10336	2.177
TS_paryes	0.14135	0.10629	1.330
soundproofTRUE	0.26358	0.22668	1.163

Table 3.2: Probability estimates for fixed effects in the operation model.

	Chisq	Df	$\Pr(>\chi^2)$	
RI_identity	50.6628	2	9.971e-12	***
TS_vis	0.4198	1	0.51706	
seq_intervene	0.0596	1	0.80705	
TS_aud.bin	4.7387	1	0.02949	.
TS_par	1.7686	1	0.18355	
soundproof	1.3521	1	0.24492	

Table 3.3: Model comparison between main operation model and model without RI type as a random slope by language.

	Df	AIC	BIC	logLik	deviance	Chisq Chi	Df	Pr($> \chi^2$)
No RI slope by language	44	4499.7	4731.7	-2205.8	4411.7			
Main model	53	4831.4	5110.9	-2362.7	4725.4	0	9	1
