# Supplementary material for: Universal Principles in the Repair of Communication Problems
Source: PLoS One. 2015 Sep 16;10(9):e0136100. doi: 10.1371/journal.pone.0136100 (PMC4573759; doi:10.1371/journal.pone.0136100)
Supplement: S4 Text — (PDF) [file pone.0136100.s005.pdf]

Supplementary Information 4
Conservation model

4.1 Conservation definition

This model investigates the cost of repair sequences (repair initiation and repair solution together) relative to the trouble source turn. For a description of the variables used, see the supplementary information 'Main model: Open and Restricted repair initiators'.

Conservation (named p4 in the R file) is measured as the relative length of the trouble source compared to the insert sequence:

$$p4 = log\left(\frac{TS\_Clength}{RS\_Clength + RI\_Clength}\right) \tag{4.1}$$

4.2 Methods

Mixed effect logit modelling was used to assess the data in R [12], using packages *lme4* [13] and *languageR* [14]. The model predicts the type of repair initiator that is used given factors relating to the previous turn. The fixed effect factors were chosen based on a-priori predictions about what would affect the likelihood of using open versus restricted repair initiators.

The intercept of the model was set to reflect the least marked situation (determined by frequency, which matches intuition well). The least marked situation is an OIR from a 1PP, 'first' sequence from a dyadic conversation in an audible language with no visible nor audio trouble, no intervening material, no parallel activity, not recorded in a soundproof booth and where B gazes to A and A gazes to B.

Probability of fixed effects was assessed using Wald chi-square test using the R package *car* [16].

4.2.1 Model structure

Below is the R code for defining the main conservation model:

```
p4~ RI_identity + TS_vis + seq_intervene +
TS_aud.bin +TS_par + soundproof +
(1 + RI_identity + TS_vis + TS_aud.bin
+ TS_par+ seq_intervene| language) + # Group by language
(1 + TS_vis + TS_aud.bin + TS_par
+ seq_intervene | recording) + # Group by recording
(1 | language.family)  # Group by language family
```

Baseline model:

```
p4~ TS_vis + seq_intervene +
TS_aud.bin + TS_par + soundproof +
(1 + RI_identity + TS_vis + TS_aud.bin
+ TS_par+ seq_intervene | language) + # Group by language
(1 + TS_vis + TS_aud.bin
+ TS_par + seq_intervene | recording) + # Group by recording
(1 | language.family)  # Group by language family
```

Null model:

```
p4 ~ 1 + (1 + RI_identity + TS_vis + TS_aud.bin
      + TS_par + seq_intervene | language)
      + (1 + TS_vis + TS_aud.bin + TS_par + seq_intervene | recording)
      + (1 | language.family)
```

Model without random intercept by language:

```
p4 ~ RI_identity + TS_vis + seq_intervene + TS_aud.bin + TS_par +
  soundproof + (0 + RI_identity + TS_vis + TS_aud.bin + TS_par +
  seq_intervene | language) + (1 + TS_vis + TS_aud.bin + TS_par +
  seq_intervene | recording) + (1 | language.family)
```

4.3 Results

4.3.1 Descriptive results

Table 4.1 show the mean length of turns by RI type

	O	R1	R2
Mean length of trouble source	21.51	29.18	28.81
Mean length of repair initiator	3.75	10.45	13.02
Mean length of repair solution	16.71	16.35	10.75

Table 4.1: Mean length of turns (in characters) by RI type.

4.3.2 Model Results

The model converged with the following fit: AIC BIC. More details on the model can be found by loading the R data file *p4_modelP4.rd*.

	Estimate	Std. Error	t value
(Intercept)	0.21	0.09	2.28
RI_identityR1	-0.05	0.09	-0.57
RI_identityO	-0.16	0.08	-2.06
TS_visyes	0.06	0.12	0.48
seq_interveneyes	-0.07	0.10	-0.68
TS_aud.binTRUE	-0.15	0.08	-1.96
TS_paryes	-0.01	0.08	-0.08
soundproofTRUE	-0.20	0.17	-1.16

Table 4.2: Fixed effects for the conservation model. Columns indicate variable, the estimated coefficient (logit scale), the standard error and the t value.

	Chisq	Df	Pr(>Chisq)
RI_identity	4.30	2.0	0.116
TS_vis	0.23	1.0	0.631
seq_intervene	0.46	1.0	0.496
TS_aud.bin	3.82	1.0	0.051
TS_par	0.01	1.0	0.935
soundproof	1.34	1.0	0.248

Table 4.3: Probability values for fixed effects (Wald chi-square test).

4.4 Universality

4.4.1 Baseline vs main

Adding RI identity does not significantly improve the fit of the model.

	Df	AIC	BIC	logLik	deviance	Chisq	Chi Df	Pr(>Chisq)
m.p4.baseline	51	3895.6	4164.5	-1896.8	3793.6			
m.p4	53	3895.3	4174.7	-1894.6	3789.3	4.3469	2	0.1138

4.4.2 Null vs Baseline

Model is not significantly improved by adding fixed effects.

	Df	AIC	BIC	logLik	deviance	Chisq	Chi Df	Pr(>Chisq)
m.p4.null	46	3892.4	4134.9	-1900.2	3800.4			
m.p4.baseline	51	3895.6	4164.5	-1896.8	3793.6	6.7784	5	0.2376

4.4.3 No random intercept by language

Model fit does not change when adding random intercept by language.

	Df	AIC	BIC	logLik	deviance
m.p4.noRandomInterceptByLang	53	3895.3	4174.7	-1894.6	3789.3
m.p4	53	3895.3	4174.7	-1894.6	3789.3

4.5 Permutation tests

The models above suggested that the average conservation was very close to 1:1. That is, the length of the trouble source turn was equal to the combined length of the repair initiator turn and the response turn. This is striking, given that the expected conservation for 3 randomly chosen turns would be 1:2. In order to assess the statistical significance of this finding, permutation tests were used. In all permutation tests below, data was only permuted within languages.

The first permutation test (reported in the main paper) tried to estimate the average conservation in normal sequences by looking only at trouble source turn lengths. The mean conservation was calculated using one turn to represent the 'trouble source' and two turns to represent the 'insert sequence'. In each permutation, 1 third of the data was randomly assigned to be 'trouble source' turns, and the remaining 2 thirds were assigned to be 'insert sequences'. The trouble source and insert sequences were randomly paired, and the $p4$ measure was calculated for

each pairing. A measure of distance from 1:1 was calculated for N pairs sequences, called $d = |\frac{\sum_{i=1}^{N} p4}{N}|$. This calculates the mean value of p4 for the sample, and calculates the distance from zero (p4 is on a log scale, so zero is equal to a ratio of 1:1). That is, d is a measure of the distance from a ratio of 1:1. d was calculated for many permutations to give a distribution of mean $p4$ values. This was then compared to d calculated for the actual data.

For the actual, unpermuted data, $d = 0.034$. 100,000 permutations were carried out and the mean d was 0.556 (mean conservation of 1:1.74, closer to the length of the insert sequence being 2 times the length of the trouble source). See the section below on why this isn't 1:2. No permutations produced a d smaller than the actual d, so the probability of the real conservation measure being close to 1:1 by chance is less than $\frac{1}{100000} = 0.00001$.

The second permutation test permuted all turn lengths (including repair initiator and response turns) so that turn lengths were randomly assigned as being the trouble source, repair initiator and response. 100,000 permutations were carried out and the mean d for permutations was 0.105. No permutations produced a d smaller than the actual d, so the probability of the real conservation measure being close to 1:1 by chance is less than $\frac{1}{100000} = 0.00001$.

A third permutation test was done, permuting only the repair initiator turns. The mean d for permutations was 0.098. 2 out of 100,000 permutations resulted in a d smaller than the actual d, so the estimated probability of the conservation measure being close to 1:1 by this more conservative measure of chance is 0.00002.

Note that permuting only the trouble source length is not meaningful, since the mean of the permuted numerator is mathematically invariant, and would be equal to the actual mean of the unpermuted data.

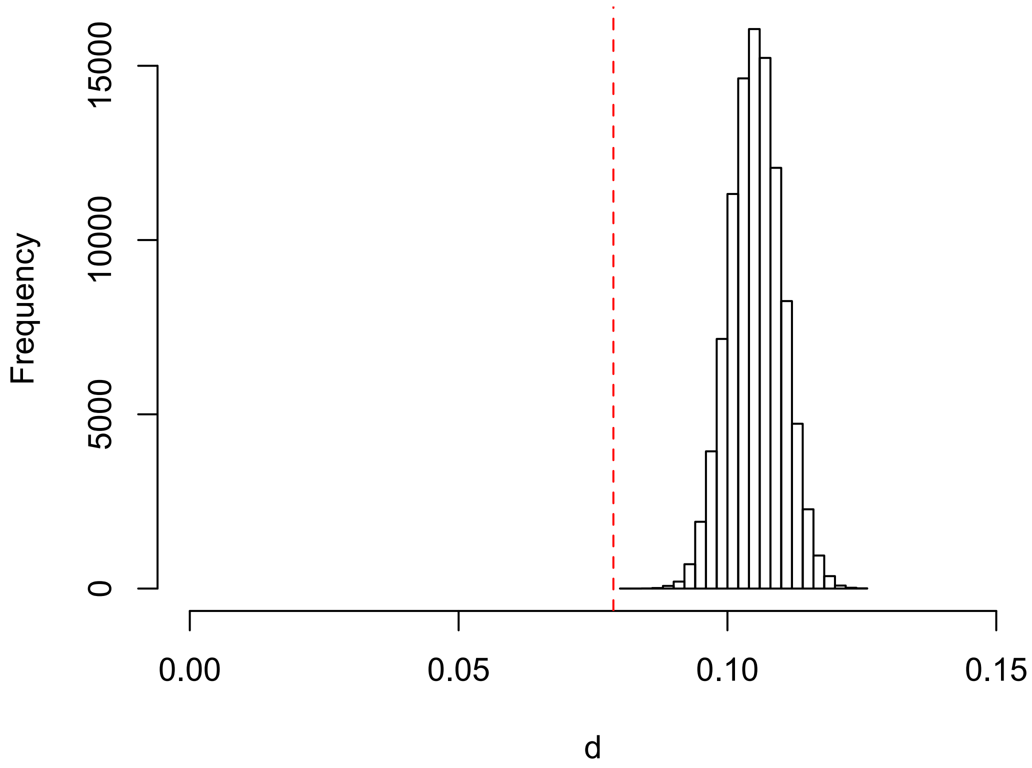

Figure 4.1: The distribution of 100,000 permuted d values, with a vertical red line representing the actual d value.

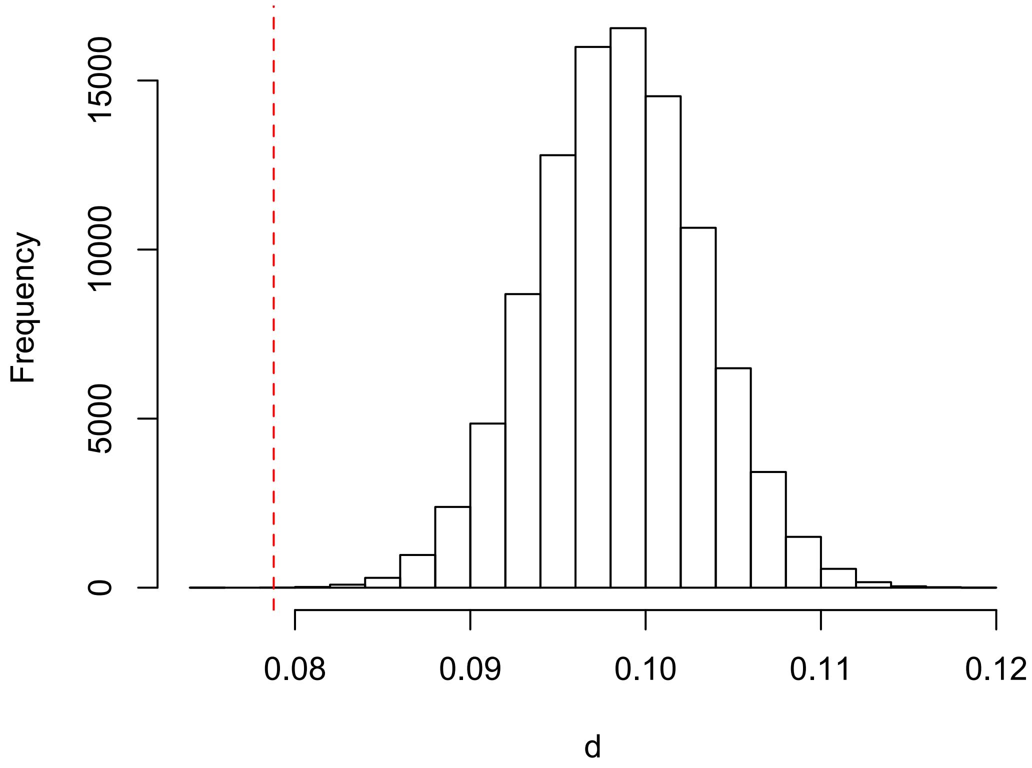

Figure 4.2: The distribution of d values for 100,000 permutations (permuting the repair initiator length only), with a vertical red line representing the actual d value.

4.6 Why isn't the permuted conservation equal to 1:2?

If all trouble source turns had equal length, then the permutation test above should have resulted in a mean conservation of 1:2. However, the mean value for the permuted data is not exactly 1:2 because the distribution of turn lengths is skewed towards short turns (see figure 4.3). This means that a short turn is more likely to be selected as one of the two insert sequence turns than the trouble source.

Figure 4.3 shows the results of simulating the permutation test. A random normal distribution is generated (30,000 points), then skewed by raising the values to a power p. When $p = 1$ and the distribution is normal, the ratio of one 'turn' compared to another two is 1:2. As the skewness increases, the ratio drops. The red triangle in the figure shows the value for the actual data. While the direction of the relationship is constant, the exact relationship between the skewness and the expected conservation is affected by the sample size and the standard deviation of the initial normal distribution. This means that the permutation test carried out above is the most appropriate method for estimating significance.

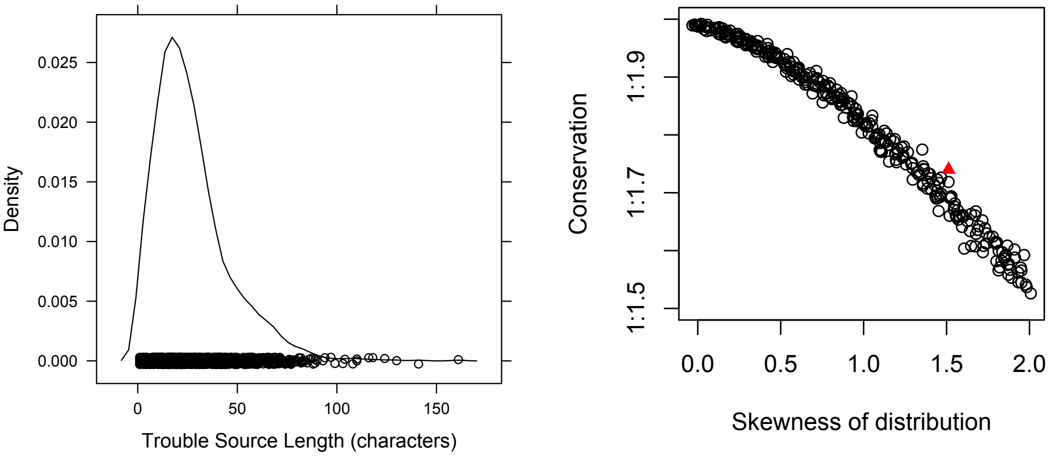

Figure 4.3: Left: The distribution of real trouble source lengths is skewed. Right: Results of simulating distributions with different skewness. The red triangle shows the values of the actual data.
