# Supplementary material for: Universal Principles in the Repair of Communication Problems
Source: PLoS One. 2015 Sep 16;10(9):e0136100. doi: 10.1371/journal.pone.0136100 (PMC4573759; doi:10.1371/journal.pone.0136100)
Supplement: S5 Text — (PDF) [file pone.0136100.s006.pdf]

Supplementary Information 5
Division of labour model

5.1 Cost

This model investigates how participants divide the labour of repair initiation and solution in the repair sequence. Participant A is the original speaker and supplier of the repair solution. Participant B is the one initiating repair. CostB is the relative cost paid by B in the insert sequence, defined as:

$$CostB = \frac{RI\_Clength}{RI\_Clength + RS\_Clength} \tag{5.1}$$

5.2 Methods

Mixed effect logit modelling was used to assess the data in R [12], using packages *lme4* [13] and *languageR* [14]. The model predicts the type of repair initiator that is used given factors relating to the previous turn. The fixed effect factors were chosen based on a-priori predictions about what would affect the likelihood of using open versus restricted repair initiators.

The intercept of the model was set to reflect the least marked situation (determined by frequency, which matches intuition well). The least marked situation is an OIR from a 1PP, 'first' sequence from a dyadic conversation in an audible language with no visible nor audio trouble, no intervening material, no parallel activity, not recorded in a soundproof booth and where B gazes to A and A gazes to B.

Probability of fixed effects was assessed using Wald chi-square test using the R package *car* [16].

5.2.1 Model structure

Below is the R code for the model structure

```
CostB.rel~
TS_Clength.logcenter +
RI_Clength.logcenter +
RI_identity + RI_identity:RI_Clength.logcenter +
oirs.per.minute +
TS_vis +
seq_intervene +
TS_aud.bin +
TS_par +
soundproof +
(1 + RI_identity + TS_vis + TS_aud.bin + TS_par+ seq_intervene
+TS_Clength.logcenter+RI_Clength.logcenter| language) + # Group by language
(1 + TS_vis + TS_aud.bin + TS_par | recording) + # Group by recording
(1 | language.family)
```

The baseline model was the same as the model above, but without a fixed effect for RI_identity. Two other models were also run - one without a random intercept by language, and one without a random slope for RI_identity by language.

5.3 Results

The model converged with the following fit: AIC = 4533.6, BIC = 4902.7, log likelihood = -2196.8. More details on the model can be found by loading the R data file *tradeoff_model.rd*.

	Estimate	Std. Error	t value
(Intercept)	0.38	0.08	4.98
TS_Clength.logcenter	-0.19	0.04	-5.15
RI_Clength.logcenter	0.76	0.10	7.66
RI_identityR1	-0.59	0.11	-5.15
RI_identityO	-0.84	0.12	-6.79
oirs.per.minute	0.22	0.12	1.75
TS_visyes	0.22	0.16	1.38
seq_interveneyes	0.04	0.10	0.40
TS_aud.binTRUE	-0.13	0.12	-1.08
TS_paryes	-0.16	0.10	-1.65
soundproofTRUE	-0.36	0.20	-1.82
RI_Clength.logcenter:RI_identityR1	-0.04	0.11	-0.39
RI_Clength.logcenter:RI_identityO	0.37	0.10	3.67

Table 5.1: Fixed effects for the tradeoff model. Columns indicate variable, the estimated coefficient (logit scale), the standard error and the t value.

Table 5.2: Probability values for fixed effects (Wald chi-square test).

	Chisq	Df	Pr(>Chisq)
TS_Clength.logcenter	26.54431297	1	2.58E-07
RI_Clength.logcenter	92.17346238	1	7.94E-22
RI_identity	73.35490475	2	1.18E-16
oirs.per.minute	3.06366932	1	0.080060687
TS_vis	1.898055147	1	0.168296172
seq_intervene	0.16083043	1	0.688393108
TS_aud.bin	1.174026785	1	0.278575387
TS_par	2.713075526	1	0.09952894
soundproof	3.298436428	1	0.069345862
RI_Clength.logcenter:RI_identity	16.62664608	2	0.000245228

5.4 Universality

The main tradeoff model is compared with models without random slopes or intercepts. In Argentine Sign Language (LSA), the operationalisation of length is indirect due to the difficulty of relating visible bodily signs to an orthographic code (see also SI 6). Although the pattern in this language looks special, it is not statistically different.

5.4.1 Baseline vs main

Adding RI identity significantly improves the fit of the model.

	Df	AIC	BIC	logLik	deviance	Chisq	Chi Df	Pr(>Chisq)	
m.tradeoff.baseline	68	4551.8	4910.4	-2207.9	4415.8				
m.tradeoff	70	4533.6	4902.7	-2196.8	4393.6	22.216	2	1.499e-05	***

5.4.2 No random intercept by language

Model is significantly improved by adding random intercept by language.

	Df	AIC	BIC	logLik	deviance	Chisq	Chi Df	Pr(>Chisq)	
m.tradeoff.noIntercept	70	4533.6	4902.7	-2196.8	4393.6				
m.tradeoff	70	4533.6	4902.7	-2196.8	4393.6	0.0076	0	< 2.2e-16	***

5.4.3 No random slope for RI identity by language

Model is not improved by adding random slope for RI identity by language.

	Df	AIC	BIC	logLik	deviance	Chisq	Chi Df	Pr(>Chisq)
m.tradeoff.noRIrandSlope	53	4510.4	4789.9	-2202.2	4404.4			
m.tradeoff	70	4533.6	4902.7	-2196.8	4393.6	10.85	17	0.8643
