# Supplementary material for: Universal Principles in the Repair of Communication Problems
Source: PLoS One. 2015 Sep 16;10(9):e0136100. doi: 10.1371/journal.pone.0136100 (PMC4573759; doi:10.1371/journal.pone.0136100)
Supplement: S6 Text — (PDF) [file pone.0136100.s007.pdf]

Supplementary Information 6
Estimating Utterance length

6.1 Orthographic length

Utterance length is used as a measure at several points in this study, for instance to test hypotheses about the different operations made relevant by repair initiation types and about the relative effort provided by the participants in the repair sequence. Therefore, some measure of the amount of content in an utterance is needed. We use the orthographic length of the transcription as a proxy for utterance length. This has also been used in other work (e.g. [17]).

The number of alphanumeric characters was calculated for each transcription (excluding punctuation and spaces). Measures for transcripts in Mandarin were taken from pinyin orthography. The transcripts for LSA were slightly different, since they code manual signs with English gloss words. The number of coded signs (orthographic words in the transcript) was used as a proxy. However, these tended to be much lower on average than for other languages, so they were scaled to have the same mean and standard deviation as the rest of the data, preserving the variation in utterance length. In interpreting the results for LSA it is worth keeping in mind that the measure for length is at least two degrees more indirect than for other languages.

Orthographic length is not directly linked to linguistic reality, but may be a good proxy for three reasons. First, it is not affected by speech rate, while a measure of time would be. Secondly, it normalises length between speakers at least within languages. Finally, it is a measure that can be easily extracted from our current data for all 12 languages.

6.2 Correlations with other measures of utterance length

Orthographic length is a surprisingly good proxy for phonetic length. Even for languages that are thought to have poor phonetic spelling, like English, orthographic length and phoneme length are highly correlated. For 160,595 English words from the CELEX database [18], orthographic length and phoneme length are significantly correlated ($r = 0.81$, $t = 557$, $p < 2\text{x}10^{-16}$). Similar correlations are found between orthographic length and syllable length ($r = 0.66$, $t = 349$, $p < 2\text{x}10^{-16}$) and between phoneme length and syllable length ($r = 0.88$, $t = 746$, $p < 2\text{x}10^{-16}$).

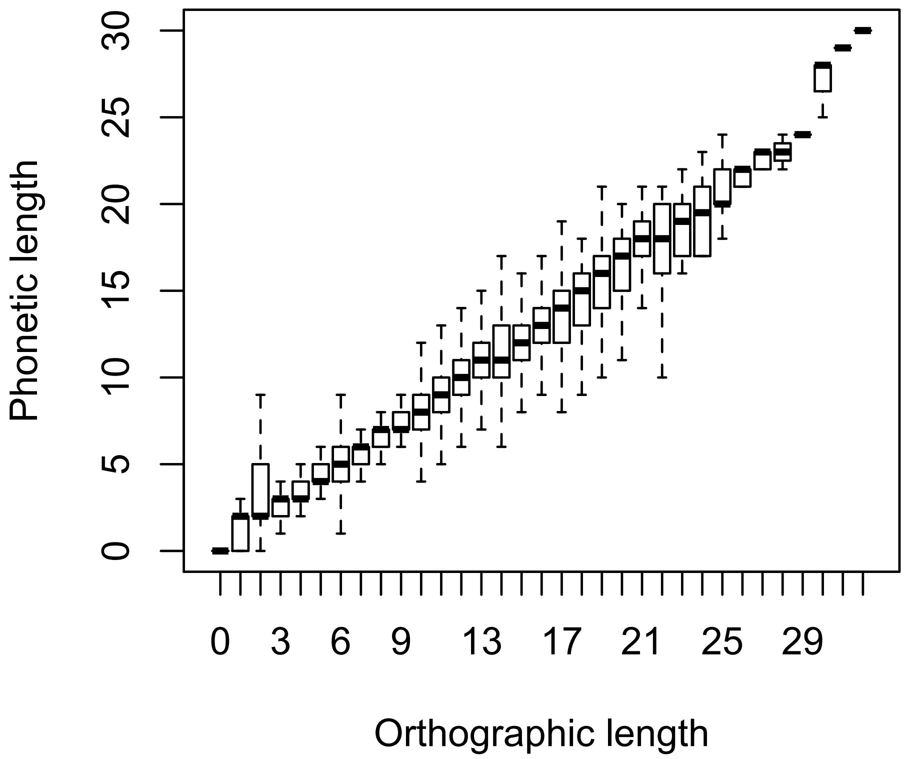

Figure 6.1: Correlation between orthographic length and phonetic length for English words from the CELEX database.

For two of the languages in the sample (English and Siwu), we also extracted the amount of time for each turn (for all turn types, not just repairs). We found that orthographic length is correlated to an even greater extent with the amount of time a turn takes to utter. In the English language data from this study, orthographic length and turn duration are highly correlated (r = 0.92, df = 9230, t = 227.9, $p < 2x10^{-16}$). Similar results were found for the Siwu data (r = 0.79, df = 7896, t = 113.31, p-value $< 2x10^{-16}$).

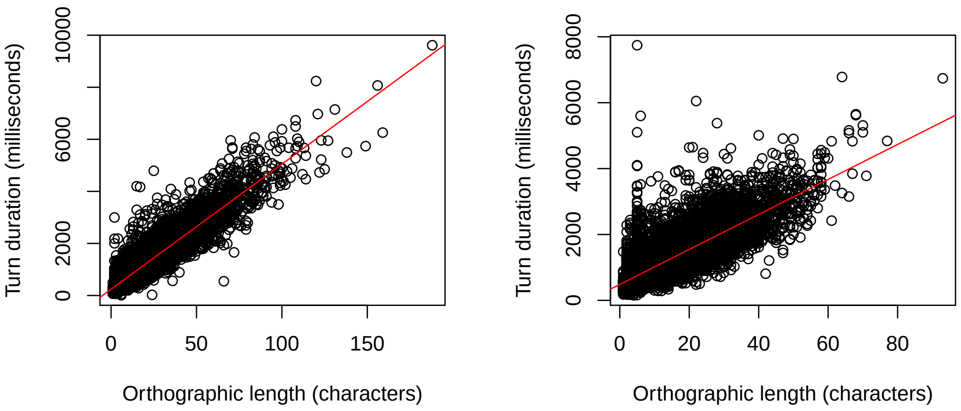

Figure 6.2: Correlation between utterance length and orthographic transcription length for English data (left) and Siwu data (right).

Bibliography

[1] American Anthropological Association (2004). American Anthropological Association Statement on Ethnography and Institutional Review Boards. [-]

[2] Linguistic Society of America (2009). Linguistic Society of America Ethics Statement. [-]

[3] Stivers, T., Enfield, N. J., Brown, P., Englert, C., Hayashi, M., Heinemann, T., Hoymann, G., Rossano, F., de Ruiter, J. P., Yoon, K.-E., and Levinson, S. C. (2009). Universals and cultural variation in turn-taking in conversation. *Proceedings of the National Academy of Sciences*, 106(26):10587–10592. [-]

[4] San Roque, L., Kendrick, K., Norcliffe, E., Brown, P., Defina, R., Dingemanse, M., Dirksmeyer, T., Enfield, N. J., Floyd, S., Hammond, J., Rossi, G., Tufvesson, S., van Putten, S., and Majid, A. (2015). Vision verbs dominate in conversation across cultures, but the ranking of non-visual verbs varies. *Cognitive Linguistics*. [-]

[5] Dingemanse, M. and Enfield, N. J. (2015). Other-initiated repair across languages: towards a typology of conversational structures. *Open Linguistics*, 1:98–118. [2]

[6] Dingemanse, M., Kendrick, K. H., and Enfield, N. J. (forthcoming). A coding scheme for other-initiated repair across languages. *Open Linguistics*. [-]

[7] , Bates, D., Kliegl, R., Vasishth, S., and Baayen, HH (submitted). Parsimonious mixed models *arXiv preprint* arXiv:1506.04967. [6]

[8] Barr, D. J., Levy, R., Scheepers, C., and Tily, H. J. (2013b). Random effects structure for confirmatory hypothesis testing: Keep it maximal. *Journal of Memory and Language*, 68(3):255–278. [6, 8]

[9] Schegloff, E. A., Jefferson, G., and Sacks, H. (1977). The Preference for Self-Correction in the Organization of Repair in Conversation. *Language*, 53(2):361–382. [2]

[10] Hayashi, M., Raymond, G., and Sidnell, J., editors (2013). *Conversational Repair and Human Understanding*. Number 30 in Studies in Interactional Sociolinguistics. Cambridge University Press, Cambridge. [2]

[11] Dingemanse, M. and Floyd, S. (2014). Conversation across cultures. In Enfield, N. J., Kockelman, P., and Sidnell, J., editors, *The Cambridge handbook of linguistic anthropology*, pages 447–480. Cambridge University Press. [5]

[12] R Core Team (2011). R: a language and environment for statistical computing. vienna, austria: R foundation for statistical computing; 2012. *Open access available at: http://cran. r-project. org*. [8, 25, 34]

[13] Bates, D., Maechler, M., and Bolker, B. (2011). *lme4: Linear mixed-effects models using S4 classes*. Online `http://CRAN.R-project.org/package=lme4`. R package version 0.999375-42. [8, 25, 34]

[14] Baayen, R. H. (2011). *languageR: Data sets and functions with "Analyzing Linguistic Data: A practical introduction to statistics"*. Online `http://CRAN.R-project.org/package=languageR`. R package version 1.4. [8, 25, 34]

[15] Schielzeth, H. and Forstmeier, W. (2009). Conclusions beyond support: overconfident estimates in mixed models. *Behavioral Ecology*, 20(2):416–420. [8]

[16] Fox, J. and Weisberg, S. (2011). *An R Companion to Applied Regression*. Sage, Thousand Oaks CA, second edition. [26, 34]

[17] Piantadosi, S., Tily, H., and Gibson, E. (2011). Word lengths are optimized for efficient communication. *Proceedings of the National Academy of Sciences*, 108(9):3526–3529. [39]

[18] Baayen, R., Pipenbrock, R., and Gulikers, L. (1995). *The CELEX lexical database (Version 2) [CD-ROM]*. University of Pensylvania, Linguistic Data Consortium. [40]
